# PAI-1 Overexpression in Valvular Interstitial Cells Contributes to Hypofibrinolysis in Aortic Stenosis

**DOI:** 10.3390/cells12101402

**Published:** 2023-05-16

**Authors:** Magdalena Kopytek, Michał Ząbczyk, Piotr Mazur, Anetta Undas, Joanna Natorska

**Affiliations:** 1 Thromboembolic Disorders Department, Institute of Cardiology, Jagiellonian University Medical College, 80 Pradnicka St., 31-202 Krakow, Poland; m.kopytek@szpitaljp2.krakow.pl (M.K.); michalzabczyk@op.pl (M.Z.); anetta.undas@uj.edu.pl (A.U.); 2 Krakow Centre for Medical Research and Technologies, John Paul II Hospital, 80 Pradnicka St., 31-202 Krakow, Poland; 3 Department of Cardiovascular Surgery, Mayo Clinic, 200 First St. SW, Rochester, MN 55905, USA; 4 Department of Cardiovascular Surgery and Transplantology, Institute of Cardiology, Jagiellonian University Medical College, 80 Pradnicka St., 31-202 Krakow, Poland

**Keywords:** aortic stenosis, fibrinolysis, LDL, nuclear factor kappa B, plasminogen activator inhibitor 1, valve interstitial cells

## Abstract

Aortic stenosis (AS) is associated with hypofibrinolysis, but its mechanism is poorly understood. We investigated whether LDL cholesterol affects plasminogen activator inhibitor 1 (PAI-1) expression, which may contribute to hypofibrinolysis in AS. Stenotic valves were obtained from 75 severe AS patients during valve replacement to assess lipids accumulation, together with PAI-1 and nuclear factor-κB (NF-κB) expression. Five control valves from autopsy healthy individuals served as controls. The expression of PAI-1 in valve interstitial cells (VICs) after LDL stimulation was assessed at protein and mRNA levels. PAI-1 activity inhibitor (TM5275) and NF-κB inhibitor (BAY 11-7082) were used to suppress PAI-1 activity or NF-κB pathway. Clot lysis time (CLT) was performed to assess fibrinolytic capacity in VICs cultures. Solely AS valves showed PAI-1 expression, the amount of which was correlated with lipid accumulation and AS severity and co-expressed with NF-κB. In vitro VICs showed abundant PAI-1 expression. LDL stimulation increased PAI-1 levels in VICs supernatants and prolonged CLT. PAI-1 activity inhibition shortened CLT, while NF-κB inhibition decreased PAI-1 and SERPINE1 expression in VICs, its level in supernatants and shortened CLT. In severe AS, valvular PAI-1 overexpression driven by lipids accumulation contributes to hypofibrinolysis and AS severity.

## 1. Introduction

Aortic stenosis (AS) is the most common acquired valvular heart disease in the elderly population from industrialized countries with no available pharmacological treatment to slow down or inhibit the disease progression [1]. The pathomechanism of aortic valve calcification is a complex and tightly regulated process, associated with activation of multiple molecular pathways. It involves accumulation of lipoproteins, chronic inflammation and activation of the coagulation system [2,3]. These actions lead to stimulation of valvular interstitial cells (VICs), playing a pivotal role in valvular calcification [4]. AS patients are also characterized by hypofibrinolysis, expressed as prolonged clot lysis time (CLT). CLT provides information about overall plasma fibrinolytic capacity, reflecting the simultaneous effect of procoagulant and profibrinolytic factors [5]. Impaired fibrinolysis was shown to be positively associated with AS severity and the thickness of fibrin deposited within stenotic valves [6,7]. However, the role of the fibrinolytic system in the development and progression of AS is not yet understood. The expression of major fibrinolytic protein—plasminogen, and its inhibitor—plasminogen activator inhibitor type 1 (PAI-1), controlling the activity of both tissue (tPA) and urokinase plasminogen activators (uPA), has been demonstrated within human stenotic aortic valves, as well as in vitro in valvular myofibroblasts cultures [8]. PAI-1 is of key importance in various chronic and acute pathophysiological processes [9], and the expression of *SERPINE1* gene encoding *PAI-1* is controlled by nuclear factor- κB (NF-κB) transcription pathway, which can be induced through pro-fibrotic signaling cascades [10]. PAI-1 secretion is strongly influenced by pro-inflammatory cytokines [11], abundantly present within stenotic aortic valves [12]. Our recent study demonstrated NF-κB expression within stenotic aortic valves as well as in VICs on both protein and mRNA levels [13]. Recently, Siudut et al. [14] showed in severe AS patients that serum lipids and apolipoproteins predicted hypofibrinolysis measured as CLT. However, it is still not clear whether impaired fibrinolysis contributes to faster AS progression or that more severe AS leads to hypofibrinolysis. Considering that LDL mediates inflammation within stenotic valves, we decided to investigate this particular lipoprotein in the context of hypofibrinolysis.

The aim of this study was to evaluate whether LDL is responsible for PAI-1 overexpression as the main factor driving hypofibrinolysis in AS.

## 2. Materials and Methods

### 2.1. Patients

We enrolled 75 patients with symptomatic severe AS between February 2019 and January 2021. All AS patients underwent first-time elective surgical aortic valve replacement at the Department of Cardiovascular Surgery and Transplantology at the John Paul II Hospital, Krakow, Poland. Data on medical history, current treatment and demographics were collected using a standardized questionnaire. Severe AS was defined as aortic valve area (AVA) < 1 cm^2^ and/or mean transvalvular pressure gradient (PG_mean_) ≥ 40 mmHg [15] on transthoracic echocardiography. All studies were evaluated by an experienced cardiologist. Arterial hypertension was diagnosed based on a history of hypertension (blood pressure > 140/90 mmHg) or preadmission antihypertensive treatment. Hypercholesterolemia was diagnosed based on total cholesterol level ≥5.0 mM, medical records or cholesterol-lowering therapy.

The exclusion criteria for AS patients were: atherosclerotic vascular disease requiring revascularization, acute infection including infective endocarditis, rheumatic AS, diabetes mellitus, advanced chronic kidney disease, need for concomitant valvular surgery (e.g., mitral valve repair), percutaneous coronary intervention, recent (<3 months) acute coronary syndrome or cerebrovascular episode, diagnosed malignancy, and pregnancy. The valvular anatomy was confirmed intraoperatively, and patients with bicuspid valve and root/ascending aortic dilatation requiring intervention were excluded from the study. The diagnosis of atherosclerosis was based on angiographically documented coronary artery stenosis >20% diameter and such patients were excluded from the study.

### 2.2. Laboratory Analysis

Fasting venous blood was drawn from the antecubital vein between 7:00 and 9:00 AM in AS patients (before aortic valve replacement). Citrated blood (9:1 of 0.106 M sodium citrate) was centrifuged at 2500× *g* for 20 min at 20 °C, while blood drawn into EDTA or serum tubes was centrifuged at 1600× *g* for 10 min at 4 °C and stored at −80 °C until analysis. Routine laboratory assays were used to determine glucose, creatinine, lipid profile, C-reactive protein (CRP), and fibrinogen.

### 2.3. Aortic Valves Preparation

Aortic valves were collected during open heart surgery. One valvular leaflet was used for in loco analysis and one for in vitro cell cultures. The third leaflet was secured for future analysis. To decalcify the incised aortic valves, they were incubated in 15% EDTA (Sigma -Aldrich, St. Louis, MO, USA) at 4 °C for 10 days. Decalcification was confirmed by calcium determination in 6 M HCl. After treatment, valves were rinsed with phosphate buffered saline (PBS), embedded in Shandon Cryomatrix frozen embedding medium (Thermo Fisher Scientific, Kalamazoo, MI, USA) for tissue cryopreservation and cryosectioned vertically into 4.5 μm slices using a Leica CM 1520 cryostat. Transverse sections were taken from the mid and commissural areas of the leaflet and stored at −20 °C until immunostaining. The control valves (n = 5) were obtained at autopsy from apparently healthy individuals of similar age without cardiac disorders.

### 2.4. Histochemical and Immunofluorescence Staining

Valvular staining was performed on randomly selected 50 AS and 5 control valves. Lipid detection was performed using Sudan black dye followed by counterstaining with nuclear fast red solution. Immunofluorescence was conducted according to the previously described protocol [16] using primary antibodies against PAI-1 (1:500; Abcam, Cambridge, UK), tPA (1:100; Novus Biologicals, Centennial, CO, USA), α_2_-antiplasmin (1:250; Santa Cruz Biotechnology, Dallas, TX, USA), plasminogen (1:500; GeneTex, Irvine, CA, USA), fibrin degradation products (D-dimer, 1:100; Bioss Antibodies, Woburn, MA, USA) and NF-κB (p65, 1:500, Abcam) and the corresponding secondary donkey or goat antibodies conjugated with AlexaFluor 488 or 594 (Abcam) (1:1000). Double-label immunofluorescence analysis was performed for PAI-1 and NF-κB. A negative IgG isotype control was performed routinely. The percentage of immunopositive areas and the fluorescence intensity (FI) were calculated as described previously [16]. The data were analyzed by two independent investigators blinded to the sample origin. The intra- and inter-observer variability was below 7%.

### 2.5. Valve Interstitial Cells In Vitro Cultures

VICs were isolated from stenotic aortic valves as previously described [17]. Experiments were performed on VICs between their third and fifth passages. When the cells reached 90–95% confluence, they were subcultured in 6-well plates in concentration of 1 × 10^5^ for immunofluorescence staining and 2 × 10^5^ for mRNA analysis in 2 mL of cell culture medium per well. VICs cultured in a standard medium (DMEM: low glucose medium, without L-glutamine and with sodium pyruvate; Biowest, Nuaillé, France) served as a negative control. To initiate the process of calcification, VICs were cultured in a calcification medium as previously described by us [18]. To induce inflammation, VICs were cultured in the calcification medium supplemented with TNF-α (50 ng/mL; Santa Cruz Biotechnology) [18] or LDL (300 µg/mL, reflecting hyperlipidemia; Sigma-Aldrich) [19].

The VICs expression of fibrinolytic proteins was assessed using immunofluorescence, as described above.

Mechanistic experiments were performed to suppress PAI-1 activity or to inhibit NF-κB transcription pathway. PAI-1 inhibitor—TM5275 (MedChemExpress LLC, Monmouth Junction, NJ, USA), which converts PAI-1 to its inactive form—was added 30 min before LDL (final concentration, 100 µM) [20]. To inhibit NF-κB, BAY 11-7082 (Sigma-Aldrich, final concentration, 10^−6^ M) was added 30 min before TNF-α or LDL stimulation [13,21].

All VICs were cultured for 7 days. The number of immunopositive cells was quantified per 100 consecutive cells per slide and 3 slides per each condition. Each experiment was repeated three times using VICs isolated from randomly selected stenotic valves.

VICs supernatants were collected after each experiment and stored at −20 °C until analysis. PAI-1 concentration in supernatants was assayed quantitatively using commercial ELISA kit (Hyphen Biomed, Neuville-sur-Oise, France) in accordance with manufacturer’s instructions.

### 2.6. CLT in VICs Supernatants

Modified CLT was performed based on Pieters method [22] using a mixture of human PAI-1-deficient plasma (25 µL; Innovative Research, Novi, MI, USA) and 5 µL of supernatants containing VICs-released PAI-1. Supernatants were diluted 1:5 due to the fact that undiluted supernatants from VICs treated with pro-inflammatory factors prolonged CLT > 300 min, which is considered as the upper limit of detection. To remove cell debris from VICs, supernatant samples were centrifuged at 1000× *g* for 5 min. Briefly, 30 µL mixture of human PAI-1 deficient plasma and VICs supernatants was mixed with 15 µM phospholipid vesicles (Rossix, Mölndal, Sweden), 15 mM CaCl_2_, 20 ng/mL tPA (Boehringer Ingelheim, Ingelheim am Rhein, Germany) and 0.5 U/mL human thrombin (Merck, Darmstadt, Germany). The turbidity was measured at 405 nm, at 37 °C. CLT was defined as the time from the midpoint of the clear-to-maximum-turbid transition to the midpoint of the maximum-turbid-to-clear transition. The experiment was repeated three times using VICs supernatants from other cell cultures. All samples were tested in triplets. Intra-assay and inter-assay coefficients of variation were <8%.

### 2.7. Relative Quantification of Transcripts by Real-Time PCR

A total of 400 ng of VICs RNA was reverse transcribed to single strand cDNA using High Capacity RNA-to-cDNA Master Mix (Applied Biosystems, Foster City, CA, USA) according to manufacturer’s instruction. The cDNA was amplified with TaqMan Gene Expression Assays (Hs00167155_m1 for PAI-1, Gene Symbol: *SERPINE1*) containing both primers and probe on an ABI PRISMR 7900HT Fast Real-Time PCR System (Applied Biosystems). Beta-actin (Hs99999903_m1, human ACTB Endogenous Control FAM/MGB Probe, Non-Primer Limited; Applied Biosystems) was used as a housekeeping gene. The comparative threshold cycle method (R = 2^−ΔΔCt^) was applied to analyze the obtained data.

### 2.8. Statistical Analyses

All statistics were performed using the STATISTICA software (Version 13.3, TIBCO Software, Palo Alto, CA, USA). Categorical variables were presented as numbers and percentages, while continuous variables were expressed as mean ± standard deviation (SD) or median and interquartile range [IQR]. Categorical variables were analyzed by Pearson’s χ^2^ or two-tailed Fisher’s exact test. Normality was analyzed by the Shapiro–Wilk test. Differences between the groups were compared using the Student’s *t*-test or Mann–Whitney U test, as appropriate. Analysis of variance (ANOVA) was used to compare continuous variables between multiple groups. Post-hoc comparisons were performed with the Tukey–Kramer HSD test. Associations between variables were calculated using Pearson’s or Spearman’s correlation coefficients, as appropriate. *p*-value < 0.05 was considered statistically significant.

## 3. Results

### 3.1. Patients’ Characteristics

Baseline characteristic of AS patients is shown in Table 1. Most of the AS patients were treated with angiotensin converting enzyme inhibitors, beta-blockers and acetylsalicylic acid. Due to concomitant atrial fibrillation, 12 (16%) AS patients were taking non-vitamin K antagonist oral anticoagulants (NOACs), with an average duration of treatment less than 2 years (19.8 ± 11.8 months) (Table 1). Of note, AS patients not receiving statin treatment (n = 18) compared to those treated with statins were characterized by increased concentrations of LDL cholesterol (3.2 [2.7–4.2] mmol/L vs. 2.3 [1.9–2.9] mmol/L, *p* = 0.0013).

### 3.2. In Loco Studies

**Figure 1:**
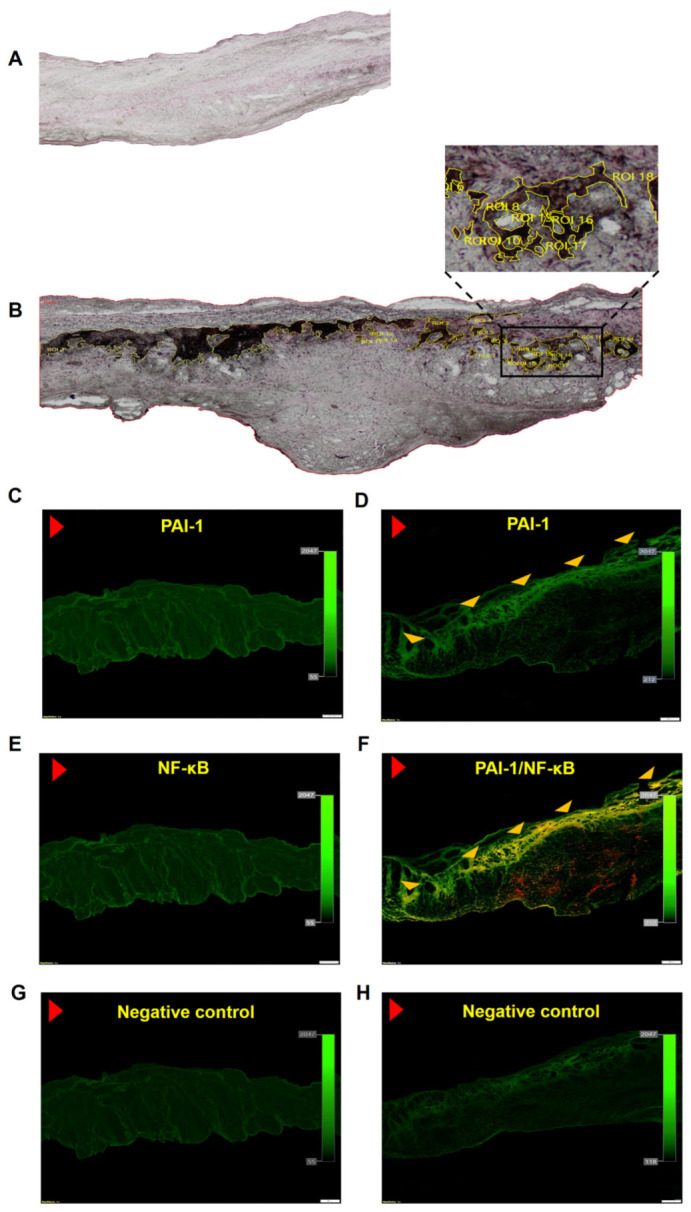
Valvular lipids accumulation together with plasminogen activator inhibitor 1 (PAI-1) and nuclear factor-κB (NF-κB) expression. Representative microphotographs of lipids accumulation in (**A**) control valve and (**B**) stenotic valve, original magnification 4×. Regions of interest (ROI) are marked in yellow. Valvular expression of (**C**) PAI-1 in control leaflets, (**D**) PAI-1 in stenotic leaflets. (**E**) NF-κB in control leaflets, and (**F**) colocalization (yellow) of PAI-1 (green) and NF-κB (red). IgG isotype control for healthy (**G**) and stenotic (**H**) valves. Red arrowhead indicates aortic side of the leaflet; yellow arrowheads indicate the immunopositive areas. Scale bar 200 μm, original magnification 4×.

**Figure 2:**
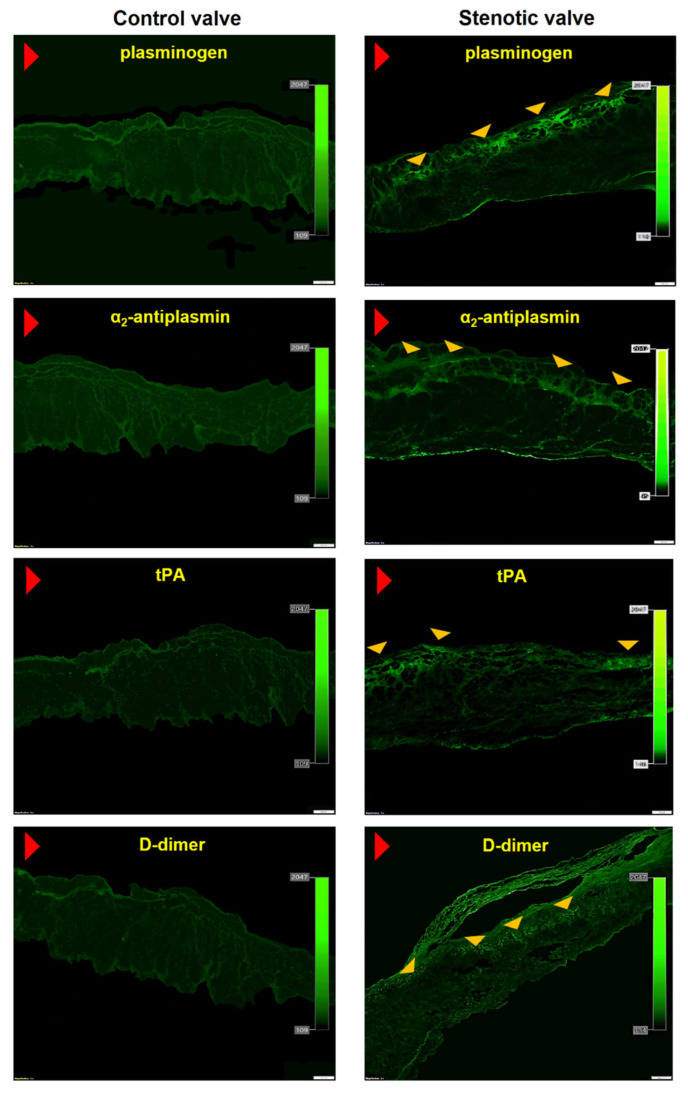
Valvular expression of fibrinolytic proteins and inhibitors. Representative microphotographs showing valvular expression of plasminogen, α_2_-antiplasmin, tissue plasminogen activator (tPA), and D-dimer in control (left panel) and stenotic aortic leaflets (right panel). Red arrowhead indicates aortic side of the leaflet; yellow arrowheads indicate the immunopositive areas. Scale bar 200 μm, original magnification 4×.

**Figure 3:**
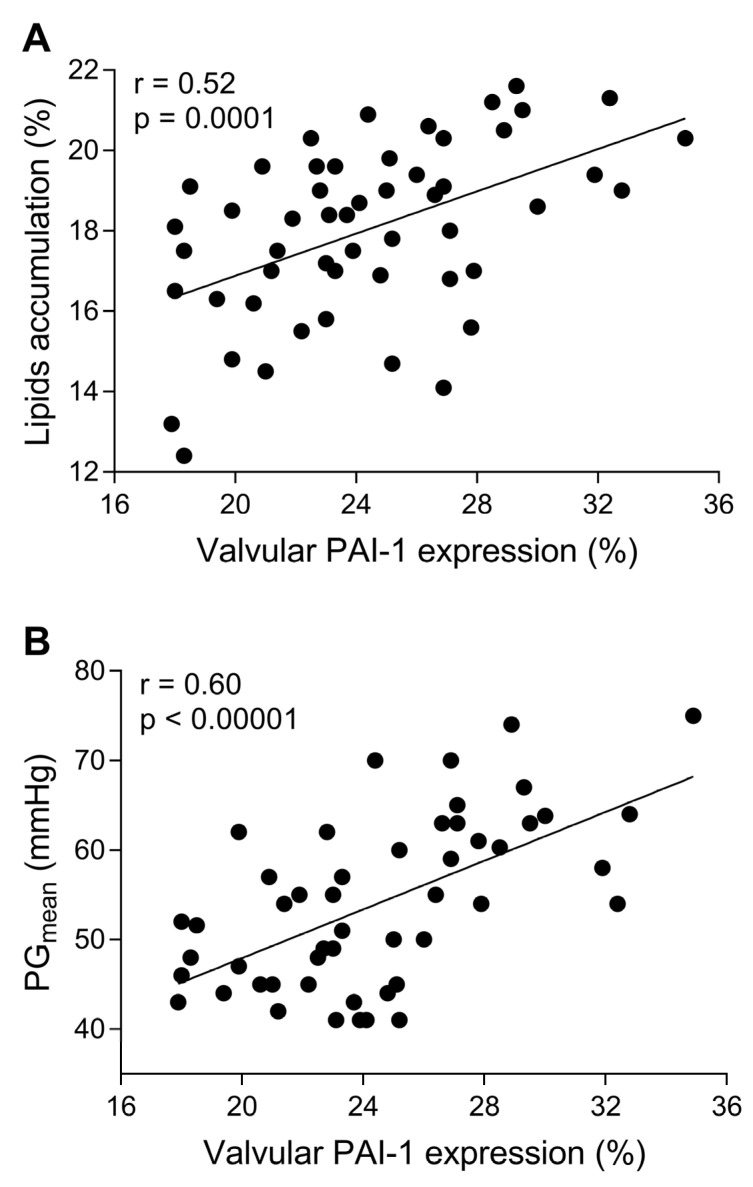
Valvular PAI-1 correlated with lipids accumulation and AS severity. The scatterplots showing associations between (**A**) valvular PAI-1 expression and lipids accumulation, (**B**) valvular PAI-1 expression and mean transvalvular pressure gradient (PG_mean_). Associations between continuous variables were calculated using Pearson’s or Spearman’s correlation coefficients. N = 50, *p*-values of <0.05 were considered statistically significant.

Massive intracellular lipid accumulation was observed within stenotic aortic valves (18.3 ± 2% of immunopositive area), but not in control leaflets (Figure 1A,B). Valvular expression of all studied fibrinolytic proteins and their inhibitors, along with D-dimer, was detected within stenotic aortic leaflets, mainly on the aortic side of the leaflets, but not in control valves (Figure 1 and Figure 2). The expression of PAI-1 (24.6 ± 4.1%) was observed in the fibrosa and spongiosa layers and presented a condensed pattern of fluorescence (Figure 1D). NF-κB expression was not detected within control valves (Figure 1E), while double staining revealed 84% co-expression of valvular PAI-1 and NF-κB within stenotic valves (Figure 1F). Valvular PAI-1 expression positively correlated with lipids accumulation and AS severity measured as PGmean (Figure 3A,B). The expression of plasminogen (16.6 ± 3.9%), α_2_-antiplasmin (12.2 ± 4.1%) and tPA (8.4 ± 3.6%) was observed in the subendothelial and fibrosa layers (Figure 2). Valvular expression of D-dimer was observed in the fibrosa and partially in spongiosa layers (Figure 2). However, the pattern of fluorescence was diffused; therefore, the FI was determined instead of the positive areas percentage. Almost 200% higher FI was observed for D-dimer-positive areas (1136 ± 223 vs. 391 ± 129 FI; *p* < 0.0001).

No differences between patients treated with NOACs or NOAC naïve were found in valvular expression of the investigated proteins (for PAI-1: 22.9% vs. 24.7%, for plasminogen: 15.6% vs. 16.8%, for α_2_-antiplasmin: 11.5% vs. 12.4%, for tPA: 8.7% vs. 8.1%; all *p* > 0.05, respectively).

### 3.3. In Vitro Studies

**Figure 4:**
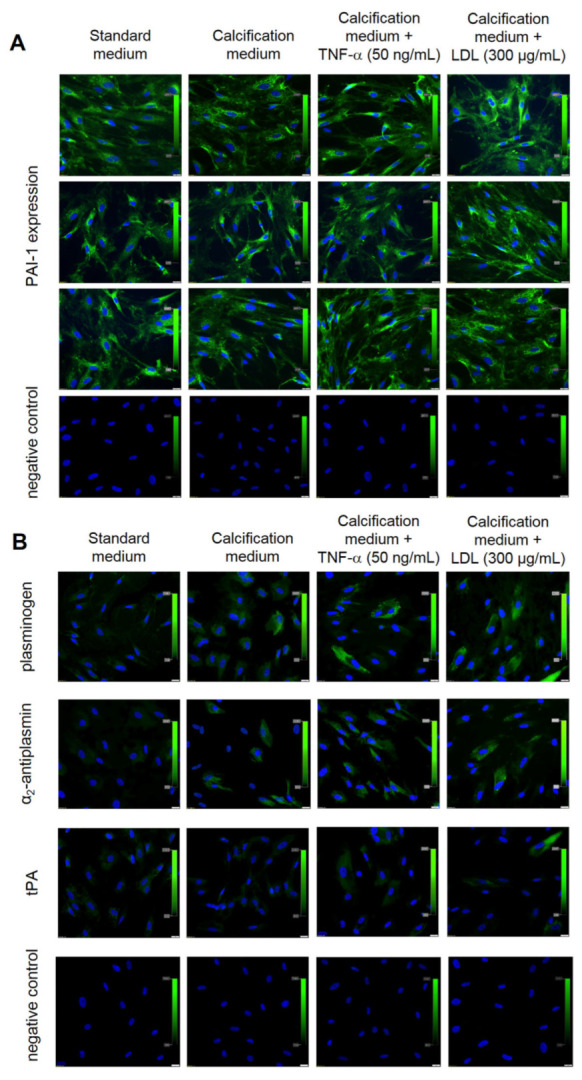
Expression of fibrinolytic proteins in valve interstitial cells (VICs) according to different culture conditions. Representative microphotographs of VICs cultured in different conditions tested for (**A**) PAI-1 expression and IgG isotype control, (**B**) plasminogen, α_2_-antiplasmin or tPA expression, and IgG isotype control. Cell nuclei are stained blue (DAPI), protein expression is green. Scale bar 20 µm, original magnification 40×. The experiment was repeated three times using VICs isolated from different valves.

Independently of culture condition, VICs showed constant expression of PAI-1 (100% of cells) (Figure 4A). VICs cultured in control or calcification medium did not express tPA or α_2_-antiplasmin, while plasminogen was poorly expressed in VICs cultured in the calcification medium (12 ± 2% of cells) (Figure 4B).

**Figure 5:**
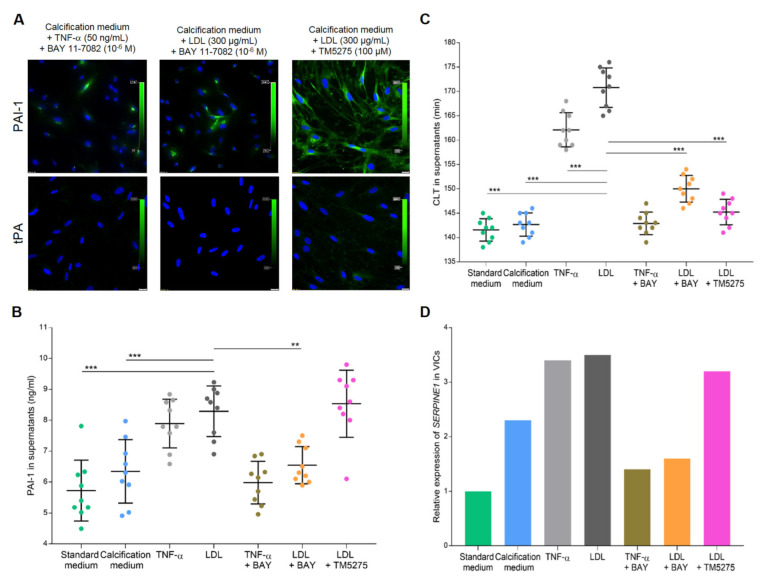
Effect of BAY 11-7082 and TM5275 on PAI-1 inhibition in VICs cultures. (**A**) Representative microphotographs of PAI-1 and tPA immunostaining in VICs stimulated with TNF−α or LDL in combination with NF−κB inhibitor (BAY 11-7082) or PAI-1 inhibitor (TM5275). Cell nuclei are stained blue (DAPI), PAI−1 is green. Scale bar 20 µm, original magnification 40×. The dot plots show (**B**) PAI−1antigen levels and (**C**) clot lysis time (CLT) in VICs supernatants from different culture conditions. Data presented as mean ± SD. ** *p* < 0.01 and *** *p* < 0.001 compared to VICs cultured in the calcification medium supplemented with LDL. Statistical analysis was performed by ANOVA and post-hoc tests. (**D**) Box plot shows relative gene expression of *SERPINE1* in VICs cultured in different conditions. Real-time PCR data are presented as mRNA expression fold change. All experiments were repeated three times using cells, supernatants or lysates from different VICs cultures; all samples were tested in triplets.

After TNF-α or LDL stimulation, 18 ± 3% or 21 ± 3% of VICs, respectively, expressed plasminogen, and 15 ± 2% or 17 ± 3% of VICs, respectively, expressed α_2_-antiplasmin but a trace expression of tPA (<10% of VICs) was observed (Figure 4B). After the use of TM5275 inhibitor, which was supposed to reduce the ability of PAI-1–tPA complex formation, the expression of tPA in VICs cultures was still at a very low level (Figure 5A).

LDL stimulation increased PAI-1 levels in VICs supernatants by 32% (*p* = 0.0005) compared to VICs cultured in the calcification medium (Figure 5B). Similarly, TNF-α stimulation increased PAI-1 concentrations in supernatants by 25% (*p* = 0.011) compared to VICs cultured in the calcification medium.

Mechanistic experiments revealed that PAI-1 expression in VICs stimulated with LDL was strongly suppressed by the NF-κB inhibitor (about 80% of cells; Figure 5A) and its concentration in VICs supernatants decreased by 22% (*p* = 0.0025; Figure 5B) compared to VICs cultured with LDL alone. The TM5275 PAI-1 inhibitor did not downregulate PAI-1 expression in VICs cultures (Figure 5A) or the level of PAI-1 antigen in VICs supernatants (*p* = 0.99; Figure 5B) compared to VICs cultured with LDL alone.

LDL stimulation prolonged CLT by 20% (*p* < 0.001) compared to calcification medium (Figure 5C). Regardless of LDL stimulation, NF-κB inhibition as well as PAI-1 activity inhibition shortened CLT by 12% and 15%, respectively, compared to VICs stimulated with LDL alone (both *p* < 0.001; Figure 5C).

Relative gene expression analysis in VICs cultures showed that stimulation with calcification medium resulted in a 1.3-fold increase in *SERPINE1* expression compared to standard medium (Figure 5D). Pro-inflammatory treatment of VICs resulted in about 2.5-fold increase in *SERPINE1* expression compared to standard medium, while NF-κB inhibition suppressed the *SERPINE1* expression by about 2-fold compared to VICs stimulated with TNF-α or LDL alone (Figure 5D). The TM5275 inhibitor treatment did not downregulate *SERPINE1* expression compared to VICs treated with LDL alone, and it was 2.2-fold higher than in VICs cultured in the standard medium (Figure 5D).

## 4. Discussion

Our study showed for the first time that, in severe AS patients, PAI-1 overexpression is driven by LDL activation of VICs. We also showed constant expression of PAI-1 in VICs, regardless of pro-inflammatory stimulation, while the expression of profibrinolytic proteins, such as plasminogen and tPA, was observed only after VICs activation. Moreover, for the first time we showed valvular α_2_-antiplasmin expression.

Valvular fibrin accumulation accounting for about 40% of the total valve area suggested impaired fibrinolysis in AS [23]. The current study reported the presence of D-dimer, a fibrin degradation product, within all valvular layers, demonstrating that fibrinolysis occurs in loco and that VICs PAI-1 overexpression contributed to hypofibrinolysis. The abundant amounts of PAI-1 with a condensed pattern of expression were found within AS valves, suggesting that this protein is synthesized de novo. Of note, a similar PAI-1 expression pattern has been reported in lung cancer [24]. We also showed for the first time expression of α_2_-antiplasmin, both within stenotic valves and in in vitro VICs cultures. The valvular expression of α_2_-antiplasmin was weak and localized subendothelially. Similarly, the α_2_-antiplasmin expression in VICs was very weak, even after the pro-inflammatory stimulation with TNF-α or LDL. It remains to be established, using quantitative methods such as RT-PCR or proteomics, whether VICs have the ability to synthesize α_2_-antiplasmin. It may be of importance to better understand VICs contribution to hypofibrinolysis in AS.

We confirmed that VICs stimulated with LDL showed enhanced *SERPINE1* expression and PAI-1 synthesis, resulting in fibrinolysis inhibition. NF-κB inhibitor suppressed PAI-1 expression on both protein and mRNA levels in VICs along with shortened CLT, regardless of pro-inflammatory stimulation with TNF-α or LDL. On the other hand, PAI-1 activity inhibition with TM5275 did not affect *SERPINE1* expression and PAI-1 antigen level but as expected promoted fibrinolysis. Therefore, we suspect that LDL may contribute to the imbalance between coagulation and fibrinolysis within stenotic aortic valves via inflammatory stimulation of VICs, resulting in PAI-1 overexpression. Valvular fibrin deposition, at least in part driven by limited ability to fibrin dissolution, contributes to valve dysfunction, hemodynamic disturbances, and increased shear stress, which activates NF-κB-related expression of pro-inflammatory genes [25]. Increased shear stress can also stimulate endothelial cells to release PAI-1 [26], contributing to systemic hypofibrinolysis. Alexopoulos et al. [27] observed that PAI-1 was strongly implicated in the pathogenesis of atherosclerosis, which shares similar mechanisms with AS. Moreover, in a murine model of atherosclerosis PAI-1 inhibitors, PAI-039 and MDI-2268, inhibited atherosclerosis development [28]. Our study also suggests that NF-κB pathway could also be a potential target for PAI-1 inhibition. NF-κB pathway inhibitors are now extensively tested in clinical studies for therapeutic intervention [29].

We also showed low in loco expression of plasminogen and tPA localized subendothelially and the trace expression of both proteins in VICs cultures, suggesting that they are released by valvular endothelial cells, rather than synthesized by VICs. Kochtebane et al. [8] identified small amounts of plasminogen, uPA, tPA, and PAI-1 within all the three tissue layers of human stenotic aortic valves and in myofibroblast cultures, however, with a large variability between valves. uPA was the only protein with enzymatic activity in myofibroblasts lysates [8]. The myofibroblasts expression of free tPA was not confirmed by the Western blot, and tPA was shown exclusively as a complex with PAI-1. We observed negligible amounts of free tPA in VICs cultures, even after the use of TM5275 inhibitor, which reduces the ability to form PAI-1–tPA complexes, reveal free tPA, and convert active PAI-1 to its inactive form [20]. Our data suggest that tPA synthesis by VICs is very limited and its release is associated with valvular endothelium or delivered to stenotic aortic valves with the bloodstream. In our opinion, the disproportion between expression of PAI-1 and profibrinolytic factors contributes to hypofibrinolysis in AS.

This study has several limitations. First, the group size was limited, and type II errors cannot be excluded. However, the study represents typical patients with symptomatic severe AS in clinical practice. Second, the valvular proteins expression was determined semi-quantitatively and estimations may be less precise. However, microscopic analysis was performed by two independent experienced investigators. Third, we did not assess the expression of uPA. We assessed hypofibrinolysis based on plasma CLT, which is the tPA-dependent model. tPA activation requires binding to fibrin, while uPA is fibrin independent and activates plasminogen in solution. Lastly, this study was performed in individuals with severe AS and tricuspid valves; thus, our results cannot be directly extrapolated to individuals with mild, moderate or bicuspid AS. Further studies on larger cohorts are needed to eliminate an influence of phenotypic aspects of the valve and aortic root characteristics.

## 5. Conclusions

Our data demonstrate that, in severe AS patients, PAI-1 is abundantly released by VICs, probably due to chronic valvular inflammation caused by LDL. PAI-1 overexpression leads to disturbed balance between coagulation and fibrinolysis, which may contribute to valvular dysfunction and AS progression.

## Figures and Tables

**Table 1:** Baseline characteristics of patients with aortic stenosis (AS).

Variable	AS Patients (n = 75)
Age, years	66 [60–71]
Male, n (%)	46 (61.3)
BMI, kg m^−2^	28 [25.7–30.6]
**Risk factors, n (%)**	
Arterial hypertension	67 (89.3)
Hypercholesterolemia	64 (85.3)
Current smoking	13 (17.3)
**Medications, n (%)**	
Beta-blockers	54 (72)
Acetylsalicylic acid	51 (68)
ACE inhibitors	48 (64)
Statins	57 (76)
Rivaroxaban	5 (6.7)
Apixaban	3 (4)
Dabigatran	4 (5.3)
**Echocardiographic parameters**	
Mean gradient, mmHg	50 [44–58]
Maximal gradient, mmHg	82 [74–94]
AVA, cm^2^	0.8 [0.7–0.9]
LVEF, %	60 [55–65]
**Laboratory investigations**	
Fibrinogen, g/L	3.4 ± 0.74
Creatinine, µmol/L	76 [70–92]
CRP, mg/L	2.0 [1.0–4.0]
Glucose, mmol/L	5.4 [5.0–5.6]
Total cholesterol mmol/L	4.1 [3.5–4.8]
LDL cholesterol, mmol/L	2.5 [2.0–3.3]
HDL cholesterol, mmol/L	1.6 [1.3–1.7]
Triglycerides, mmol/L	1.1 [0.9–1.7]

Data presented as numbers (percentages), mean ± standard deviation or medians [interquartile range]. Abbreviations: ACE inhibitors, angiotensin converting enzyme inhibitors; BMI, body mass index; CRP, C-reactive protein; LVEF, left ventricular ejection fraction.

## Data Availability

The data presented in this study are available on request from the corresponding author. The data are not publicly available due to ongoing experiments.
